# Editorial: Chronic effects on brain development induced by early-life stress

**DOI:** 10.3389/fnins.2023.1293325

**Published:** 2023-10-18

**Authors:** Yusuke Takatsuru, Kazuya Miyagawa

**Affiliations:** ^1^Division of Multidimensional Clinical Medicine, Department of Nutrition and Health Sciences, Toyo University, Itakura, Japan; ^2^Department of Pharmacology, School of Pharmacy, International University of Health and Welfare, Ohtawara, Japan

**Keywords:** neuronal development, maternal behavior, chronic stress, mental disorders, multi-generation

Undoubtably, early-life stress (ELS) during the perinatal period causes poor brain development, and it is implicated in the onset of several psychotic disorders. ELS, including early long-term institutionalization, causes structural and functional changes in the brain (Teicher et al., 2016; Herzberg and Gunnar, 2020, Tian et al.). The number of parvalbumin–, calbindin–, and calretinin–positive neurons is decreased in both the nucleus acumens and amygdala of ELS-exposed rats, accompanied by a reduction in the size of the neuron cell body (Aleksic et al.). ELS exposure of rats alters the activation of the reward circuitry, interferes with the normal formation of context–reward associations, and disrupts the normal reward access hierarchy formation in adulthood (Ryakiotakis et al.). ELS-exposed mice show hypersensitivity and increased levels of glutamate released (Takatsuru et al., 2009; Toya et al., 2014). In the brain of ELS-exposed mice, the stability of the mushroom spine is decreased (Takatsuru et al., 2009), and the motility of microglia is increased (Takatsuru et al., 2015). Furthermore, when focusing on pregnancy, research conducted on mice suggests that excessive exposure to prenatal stress can result in an increased vulnerability to stress associated with the disruption of the development of 5-HT neurons of offspring (Miyagawa et al., 2011, 2015).

These changes are always difficult to reverse; thus, the effects of ELS last long, from childhood to old age. These structural and functional changes potentially induce several disorders; ELS is a risk factor for depression and anxiety disorders (Pervanidou and Chrousos, 2018; Juruena et al., 2020; LeMoult et al., 2020). Depression affects people from having a normal life, such as attending school, holding a job, and spending time on hobbies. Depression sometimes leads to suicide owing to a decreased quality of life. Depression is also one of the risk factors for dementia (Bennett and Thomas, 2014; Hayley et al., 2021), and ELS itself potentially induces dementia both in humans (Harris et al., 2016; Wise, 2016) and rodent models (Yajima et al., 2018). Thus, ELS disrupts human life, and the treatment of symptoms induced by ELS is important.

The effects of ELS are confirmed in not only a single generation but may sometimes extend also to the next generation. Adult women who experienced sexual or physical abuse in childhood show a disrupted hypothalamic–pituitary–adrenal axis (Heim et al., 2001). Such a disruption definitely induces undesirable maternal behavior. It has been reported that parents who have experienced childhood abuse and neglect are more likely to neglect their offspring (Widom et al., 2015). Note that this is not true for all cases; approximately 30% of victims neglect their own children. Thus, this behavioral change is not always induced by ELS. Both favorable and unfavorable environments can induce behavioral changes. Support from other people during development, perinatal care, and nursing can change the behavior of mothers who suffered from ELS. This change was also detected in a rodent model (Mitani et al., 2018). Approximately 30% of ELS-exposed mother mice show neglect behavior.

It has also been reported that the offspring of ELS-exposed humans often suffer from neurophysiological diseases even when the offspring have not been exposed to ELS (Bifulco et al., 2002; Kim et al., 2009; Heim et al., 2010). This has also been detected in a rodent model (Mitani et al., 2018). Offspring of ELS-exposed mice also showed hypersensitivity, and approximately 30% of the offspring showed neglect behavior. Note that the offspring themselves were not exposed to ELS; but were born from an ELS-exposed mother. The involvement of epigenetic factors such as the alteration of DNA methylation, which are transferred through germ cells, has been implicated (Cameron et al., 2008; Franklin et al., 2010; Heim and Binder, 2011; Weaver et al., 2014); however, the mechanism underlying the multigenerational effects of ELS has not yet been clarified. The biggest problematic issue is that victims of ELS cannot avoid its effects. They realize the effects of ELS after several disorders develop because of non-reversible brain changes. The offspring also feel miserable because they cannot choose their parents. Thus, the effects of ELS on humans are one of the most important Research Topics.

However, the effects of ELS on brain development and functions are not yet fully understood, and the treatment of ELS-related disorders remains unknown. To clarify these issues, we also consider several conditions similar to ELS, such as infants born very and extremely preterm (Cook et al.: changes in the functional architecture with increasing age of preterm infants exhibit a different trajectory relative to *in utero* fetus), central precocious puberty (Yoshii et al.: increase in thickness of the precuneus area of the right hemisphere), and the juvenile justice system (Orendain et al.). It is also helpful to study newly generated risk factors, such as e-cigarette exposure [Lee et al.: exposure to e-cigarettes alters the mammalian target of rapamycin (mTOR)C1 and mTORC2 signaling in the developing hippocampus] and combined drug effects (Rêgo et al.: the administration of fentanyl enhances hippocampal neurogenesis and anxiety without affecting spatial learning and memory in ELS rats).

The number of articles on this Research Topic is insufficient as we expected, indicating that the study of ELS is inadequate and inactive. One of the reasons for this is obtain that experiments conducted in the study of ELS take a long time to complete. Many researchers, including us, want to get results as soon as possible. However, we should keep on studying to find ways to treat the effect of ELS. We hope this Research Topic encourages many researchers to continue/begin the study of ELS.

## Author contributions

YT: Writing—original draft, Writing—review and editing. KM: Writing—review and editing.
